# Simulation Study on the Mass Transport Based on the Ciliated Dynamic System of the Respiratory Tract

**DOI:** 10.1155/2019/6036248

**Published:** 2019-11-27

**Authors:** Peng-Fei Zhu, Xiang Li, Ao Li, Yuan Liu, Duan-Duan Chen, Yuan-Qing Xu

**Affiliations:** ^1^School of Life Science, Beijing Institute of Technology, Beijing 100081, China; ^2^Key Laboratory of Convergence Medical Engineering System and Healthcare Technology, The Ministry of Industry and Information Technology, Beijing Institute of Technology, Beijing 100081, China

## Abstract

To study the mass transport of mucociliary clearance of the human upper respiratory tract, a two-dimensional mass transport model based on the ciliated movement was established by using the immersed boundary-lattice Boltzmann method (IB-LBM). In this model, different characteristics of the mucus layer (ML) and the periciliary liquid (PCL) were taken into account. A virtual elastic membrane was introduced to divide the two layers dynamically. All moving boundaries that were involved in the present simulation were modeled with the immersed boundary. The Newtonian fluid was used to model the flow in PCL, and the viscoelastic fluid based on the Oldroyd-B model was used for the flow in ML; the two types of flow were both solved by the LBM framework. Based on the model, the ML thickness, the cilia density, and the phase difference of adjacent cilia were regulated, respectively, to study the transport velocity of the ML. In addition, the motion law of solid particles in PCL was also studied. According to the results, four primary conclusions were drawn. (1) At a given beating pattern, the increase of the ML thickness will decrease its transport velocity. (2) Increasing the cilia density can promote the mean transport velocity of the ML. (3) By raising the phase difference of adjacent cilia to a certain scope, the transport of ML can be accelerated. (4) In PCL, particles initially located on the upper part of the cilia tend to migrate upward and then get close to the ML. The above study can provide some reasonable explanations for the mechanism of the mucociliary clearance system, which is also helpful to the further understanding of the mass transport principle of the human upper respiratory tract.

## 1. Introduction

Mucociliary clearance (MCC) system is an important part of the human immune system, which is responsible for the removal of bacteria, viruses, dust, and various harmful particles in the inhaled air. It plays an important role in protecting human health [1]. The MCC mainly consists of cilia and mucus attached to the respiratory tract surface. The mucus is made up of two layers, i.e., the mucus layer (ML) and the periciliary liquid (PCL). ML is a non-Newtonian fluid layer of high viscosity. It can capture and filter harmful particles in the air. PCL is a Newtonian fluid layer of less viscosity, which has similar physical properties to the water. The PCL is important to lubricate the surface of the respiratory tract and facilitate the swing of the cilia [2]. By performing a coordinated spontaneous beating, cilia promote the movement of ML to the throat and then transport foreign materials out of the body [3].

There are many factors affecting the mass transport of MCC, such as human activity, drug stimulation, pH level variation, and external environment [4]. Other studies reveal that the particulate matter 2.5 (PM2.5) can change the secretion of respiratory mucus and even destroy the structure of cilia, resulting in abnormal cilia function and dysfunction of ML transport [5]. In addition, some artificial traumas and congenital diseases, such as surgery, primary ciliary dysplasia (PCD), and immobile cilia syndrome (ICS), can affect the structure or the movement state of cilia and then affect the work efficiency of MCC [6, 7]. Therefore, the study of cilia movement mechanism is practically significant. However, up to now, the relevant experiment study is insufficient for two major reasons. The first is that the respiratory structures are complex, and the cilium contains various microscopic motion patterns. The other is that the appropriate noninvasive experimental equipment is still lacking in application.

In recent years, with the rapid development of computer science and numerical technology, computer simulation of MCC has attracted more attention and the mass transport of MCC has become an attractive topic in the fields of both physiology and physics [8]. To model the beating cilia, the immersed boundary method (IBM) is one of the most popular research methods. Peskin introduced this method for the first time to study the cardiac blood flow around the heart valve [9]. Now, it has been widely used in solving biological problems related to moving boundaries [10]. By combining IBM with the finite difference method, Lee et al. [11] established a two-dimensional (2D) model to study the underlying mechanisms of some diseases in the MCC system. They concluded that the beat frequency and density of cilia and the depth of PCL were the key factors affecting the transport velocity of the ML. Jayathilake et al. [12] studied three cilia beating patterns: windscreen wiper motion, rigid planar motion, and normal motion. It was found that abnormal cilia beating patterns, such as windscreen wiper motion and rigid plane motion, greatly reduced or prevented the transportation of ML. Moreover, Jayathilake et al. [13] developed a 3D model to simulate the movement of human lung cilia in a single layer of mucus. The results showed that thinner PCL or higher cilia beating frequency could increase the mean flow velocity of PCL. By applying the immersed boundary-lattice Boltzmann method (IB-LBM) framework, Shahmardan et al. [14] studied the effect of mucus thickness on its transport efficiency. The results showed that an increase in mucus thickness caused by air pollution and smoking reduces the mean mucus velocity. In particular, for the first time, Sedaghat et al. [15, 16] used IB-LBM to solve the two-layer mucus model. The viscoelastic flow was established with the Oldroyd-B model in the upper layer ML, and the Newtonian fluid was set in the lower layer PCL. Their study revealed that the viscosity had a significant effect on mucus transport efficiency.

Although great progress has been made in the study of the MCC system, there are still some principles in MCC that need to be discovered. On one hand, Hoffmann and Cortez [17] studied the motion of two adjacent cilia; they concluded that the speed of ML tended to increase in the out-of-phase beating. We think that, in this topic, the phase difference of adjacent cilia on ML transportation deserves further study. On the other hand, besides the cilia and flow, some floating rigid particles are usually found in MCC [18], which may be the foreign matter or come from the respiratory tract surface. Moreover, some other topics, such as the decontamination mechanism of cilia [19] and drug effect on the motion of cilia [20], are also worth further exploring.

In this study, the Oldroyd-B model was used to model the viscoelastic fluid in ML. A 2D IB-LBM was applied to simulate the transport velocity of ML in the MCC system. A virtual membrane was used to separate the two layers of ML and PCL dynamically. Compared to the model in References [15, 16], we improved the motion control of cilia. A moving virtual cilium was introduced to lead the motion of the actual cilia through elastic springs. To do this, we can obtain anticipant beating patterns of the cilia. Based on the model, by changing the thickness of ML, the density of cilia, and the phase difference of adjacent cilia, the transport efficiency of the ML was studied in detail. Moreover, we also conducted a study on the particle motion principle in PCL, where the particle size and initial position on the particle migration were discussed.

The rest of this paper is organized as follows.  Section 2 gives the physical model and the numerical method.  Section 3 verifies our models.  Section 4 exhibits and discusses the numerical results. And the final conclusions are listed in Section 5.

## 2. Models and Methods

### 2.1. Physical Model

**Figure 1:**
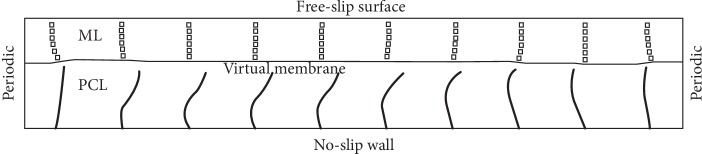
The diagrammatic sketch of MCC.

The diagrammatic sketch of the physical model is displayed in Figure 1.

The left and right boundaries of the channel are set as periodic. The top boundary is a slip wall. The bottom boundary is a nonslip wall, on which the root ends of cilia are fixed. Similar to the model in References [15, 16], the mucus is divided into two layers, ML and PCL. They are divided by a virtual elastic membrane. The fluid is viscoelastic in ML and is Newtonian in PCL.

In order to measure the transport efficiency of the ML, as shown in Figure 1, we set a group of tracepoints to float with the flow. The mean velocity of ML is estimated by calculating the mean velocity of these tracepoints within a time scope of 3*T*. Worth noting is that, in the chosen time window, the flow in ML has been fully developed.

### 2.2. Mathematical Models of Flow and Structure

In this model, the cilia are immersed in a viscous fluid; their autonomous beating motion drives the flow of the surrounding mucus. The flow is governed by the Navier–Stokes equation:

(1) $$\begin{array}{c} \rho \frac{\partial u}{\partial t}+\rho u\cdot \nabla u=-\nabla p+\mu \nabla^{2}u+F,\\ \;\nabla \cdot u=0,\; \end{array}$$

where *u* is the velocity, *μ* is the kinetic viscosity of the fluid, *p* is the pressure, and *F* is the resultant force on the cilia.

To divide ML and PCL, the cilia and the membrane consist of a set of nodes connected by springs in a consecutive way [21, 22]. The force on cilia nodes through the spring is made up of three components, the stretching force *F*_s_, the bending force *F*_b_, and the driving force *F*_c_ from the virtual cilia. Then we get the resultant force *F*=*F*_s_+*F*_b_+*F*_c_. For the membrane, each node involves two force components of *F*_s_ and *F*_b_. Then there is *F*=*F*_s_+*F*_b_. The stretching force *F*_s_ satisfies Hooke's law of elasticity in the tangent direction. It maintains the inherent length of the boundary, which can be written as

(2) $$\begin{array}{c} F_{\text{s}}\left(s,t\right)=\;\frac{\partial }{\partial s}\left[K_{\text{s}}\left(\left|\frac{\partial X\left(s,t\right)}{\partial s}\right|-1\right)\frac{\partial X\left(s,t\right)}{\partial s}\right], \end{array}$$

where *K*_s_ is the extensional coefficient.

The bending force *F*_b_ is the bending moment of the boundary in the normal direction, which is derived from the Frechet derivative of the bending energy formula based on the virtual work principle [23]. It is calculated by

(3) $$\begin{array}{c} F_{\text{b}}\left(s,t\right)=K_{\text{b}}\frac{\partial^{4}X\left(s,t\right)}{\partial s^{4}}, \end{array}$$

where *K*_b_ represents the bending coefficient. In addition, the moment and transverse stress are zero for nodes at both ends of the cilia [24]:

(4) $$\begin{array}{c} \frac{\partial^{2}X\left(s,t\right)}{\partial s^{2}}=0,\\ \frac{\partial^{3}X\left(s,t\right)}{\partial s^{3}}=0. \end{array}$$

**Figure 2:**
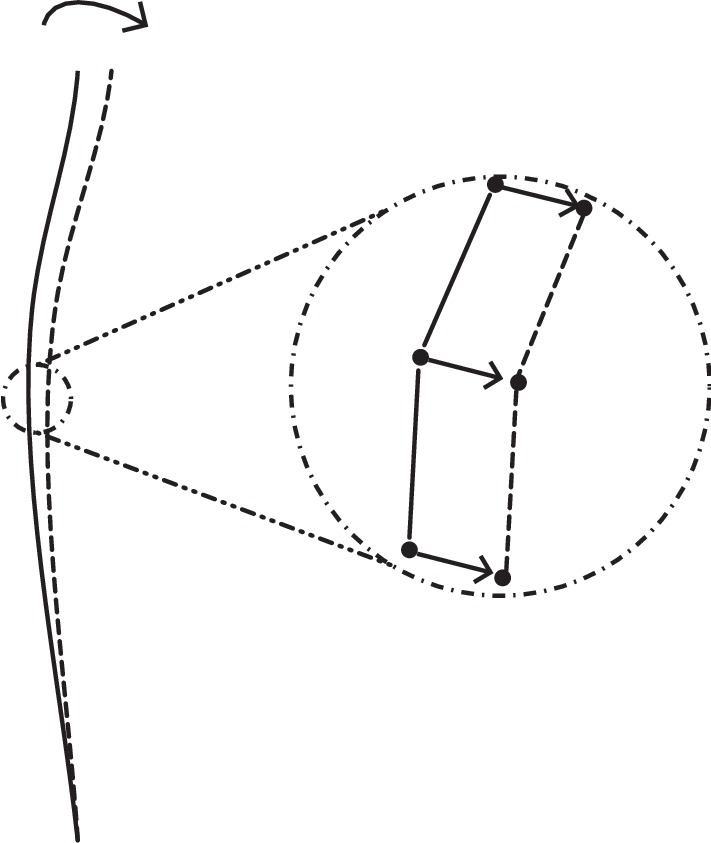
The actual cilium and virtual cilium, and their connections. The solid line represents the actual cilium, and the dashed line represents the virtual cilium. The actual cilium is driven by the virtual cilium through a group of virtual springs.

In this model, in order to realize the autonomous beat of cilia, a virtual cilium with a fixed beat posture is introduced to lead the moving of the actual cilium boundary. A group of virtual springs are used to connect the virtual and the actual cilium. They are displayed in Figure 2.

The elastic force *F*_c_ is defined as *F*_c_=*K*_sl_(*Y* − *X*), where *K*_s1_ is the corresponding elastic coefficient and *Y* represents the boundary of the virtual cilium.

A hard particle model was used in the present study, which follows Newton's second law. The velocity of the particle is made up of two components, i.e., the translational velocity *U*_p_ and the angular velocity *Ω*_p_; the corresponding forces on the particle are formulated as [25]

(5) $$\begin{array}{c} F_{\text{tra}}=m_{\text{p}}\frac{\text{d}U_{\text{p}}}{\text{d}t}=-\int_{\text{CS}}\sigma_{\text{s}}\cdot n\text{d}S+\left(\rho_{\text{p}}-\rho_{\text{f}}\right)V_{\text{p}}g,\\ F_{\text{ang}}=I_{\text{p}}\frac{\text{d}\Omega_{\text{p}}}{\text{d}t}=-\int_{\text{CS}}\left(X_{\text{s}}-X_{\text{c}}\right)\times \left(\sigma_{\text{s}}\cdot n\right)\text{d}S, \end{array}$$

where the subscripts p and f, respectively, stand for particle and fluid. *m*_p_ is the mass of the particle. *ρ* is the density. *I*_p_ is the moment of inertia. **X**_s_ − **X**_c_ stands for the position vectors of a Lagrangian point and the center of particle, respectively. CS stands for the surface of the particle, and **n** is the normal vectors of triangles. **σ**_s_ is the fluid stress tensor, and *g* is the gravitational acceleration. Moreover, the repulsive effects between two boundaries also need to be considered, including the cases of particle surface to particle surface, particle surface to cilium boundary, and particle surface to wall boundary. The repulsive force is marked with *F*_rep_; it is calculated according to the model suggested in Reference [26]. Then the total force on the particle is *F*_p_=*F*_tra_+*F*_ang_+*F*_rep_.

In the Oldroyd-B model for the viscoelastic fluid in ML, the pressure tensor **σ** is decomposed into two parts as [27]

(6) $$\begin{array}{c} \sigma_{M}=\sigma_{M,N}+\sigma_{M,E}, \end{array}$$

where **σ**_*M*,*N*_ is the part of the elastic tensor in the Newtonian fluid, and **σ**_*M*,*E*_ is the part of the elastic tensor in the non-Newtonian fluid, where **σ**_*M*,*N*_ is given as

(7) $$\begin{array}{c} \sigma_{M,N}=2\eta_{M,N}D,\; \end{array}$$

where *D* is the strain rate tensor and it is defined as

(8) $$\begin{array}{c} D=\frac{1}{2}\;\left(\nabla u+{\left(\nabla u\right)}^{T}\right). \end{array}$$

For **σ**_*M*,*E*_, it is derived from the Upper Convected Maxwell model (UCM), which is given as

(9) $$\begin{array}{c} \sigma_{M,E}+\lambda {\overset{\nabla }{\sigma }}_{M,E}=2\eta_{M,E}D, \end{array}$$

where *λ* is the relaxation time, which accounts for the magnitude of hydroelastic properties. ${\overset{\nabla }{\sigma }}_{M,E}$ is the upper material derivative of **σ**_*M*,*E*_, and it is expressed as

(10) $$\begin{array}{c} {\overset{\nabla }{\sigma }}_{M,E}=\frac{\partial \sigma_{M,E}}{\partial t}+u\cdot \nabla \sigma_{M,E}-\sigma_{M,E}\cdot \nabla u-\nabla u^{T}\cdot \sigma_{M,E}. \end{array}$$

In addition, the viscosity in this model can be divided into the Newtonian part (*η*_*M*,*N*_) and the elastic part (*η*_*M*,*E*_). The total viscosity *η*_*M*_ of the fluid is *η*_*M*_=*η*_*M*,*N*_+*η*_*M*,*E*_.

There are several methods to solve the model to get the tensor **σ**. In the present study, the method based on LBM in Reference [28] is used to solve the Oldroyd-B model.

### 2.3. The IB-LBM Framework

In our study, the D2Q9 model of the lattice Boltzmann method is employed to solve the flow. The single relaxation time lattice Boltzmann equation is [29, 30]

(11) $$\begin{array}{c} g_{i}\left(x+e_{i}\Delta t,\;t+\Delta t\right)-g_{i}\left(x,t\right)=-\frac{1}{\tau }\left[g_{i}\left(x,t\right)-g_{i}^{\text{eq}}\left(x,t\right)\right]+\Delta tG_{i}, \end{array}$$

where *g*_*i*_(*x*, *t*) is the distribution function of particles, *e*_*i*_ is the particle velocity, ∆*t* is the time step, *g*_*i*_^eq^(*x*, *t*) is the equilibrium distribution function, *τ* is the nondimensional relaxation time, and *G*_*i*_ shows the effect of volume force on the distribution function.

In equation (11),  *g*_*i*_^eq^(*x*, *t*) and *G*_*i*_ are given as [31]

(12) $$\begin{array}{c} g_{i}^{\text{eq}}=\omega_{i}\rho \left[1+\frac{e_{i}\cdot u}{c_{\text{s}}^{2}}+\frac{uu:\left(e_{i}e_{i}-c_{\text{s}}^{2}I\right)}{2c_{\text{s}}^{4}}\right],\\ G_{i}=\left(1-\frac{1}{2\tau }\right)\omega_{i}\left[\frac{e_{i}-u}{c_{\text{s}}^{2}}+\frac{e_{i}\cdot u}{c_{\text{s}}^{4}}e_{i}\right]\cdot f, \end{array}$$

where **f** is the vector of the body force density, and *ω*_*i*_ is the weight defined by *ω*_0_ = 4/9, *ω*_*i*_ = 1/9 for *i* = 1 to 4, and *ω*_*i*_ = 1/36 for *i* = 5 to 8. $C_{\text{s}}=\Delta x/\sqrt{3}\Delta t$ is the sound speed.

In this study, the immersed boundary method is applied to deal with the interaction between cilia and fluid. The equation of IBM is given by

(13) $$\begin{array}{c} f\left(x,t\right)=\int_{\Gamma }F\left(s,t\right)D\left(x-X\right)\text{d}s, \end{array}$$

where *F*(*s*, *t*) is a Lagrangian force that acts on the fluid by cilia, and Dirac's delta function *D*(*x* − *X*) is given by *D*(*x* − *X*)=*δ*(*x* − *X*)*δ*(*y* − *Y*). The velocity on cilia can be obtained by interpolation of fluid points nearby. To update its location, the velocity of cilia *U*(*s*, *t*) is given as

(14) $$\begin{array}{c} U\left(s,t\right)=\int_{\Omega }u\left(x,t\right)D\left(x-X\right)\text{d}x. \end{array}$$

## 3. The Verification of the Oldroyd-B Model

**Figure 3:**
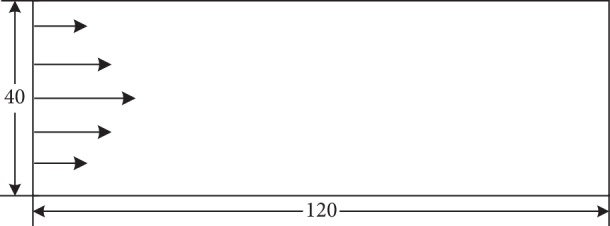
The channel model for the verification of the Oldroyd-B model.

In this section, a 2D Poiseuille flow is used to verify the numerical method for the Oldroyd-B model. The model is shown in Figure 3, in which the channel consists of two parallel planar no-slip walls. The grid-scale *L* × *H* is 120 × 40. A parabolic velocity profile is set at the inlet, and the free flow condition is set at the outlet. The nonequilibrium extrapolation scheme is utilized to model the microboundary conditions in LBM.

As the viscoelastic Poiseuille flow is fully developed, the analytical results of velocity and configuration tensors can be expressed by the following formulas:

(15) $$\begin{array}{c} u_{x}=\frac{4u_{\max}\left(L_{y}y-y^{2}\right)}{L_{y}^{2}},\\ A_{xx}=1+\frac{32\lambda^{2}u_{\max}^{2}{\left(-2y+L_{y}\right)}^{2}}{L_{y}^{4}}, \end{array}$$

where *u*_max_ is the maximum speed at the entrance, and *L*_*y*_ is the characteristic length of the geometric model, which is the height of *y*-direction. The Weissenberg number *W*_*e*_ is set as 0.01 and 1. The Reynolds number Re=4, and viscosity ratio *Rv*=*η*_*M*,*N*_/(*η*_*M*,*N*_+*η*_*M*,*E*_)=0.9. The maximum velocity at the inlet is *U*_max_=0.1.

**Figure 4:**
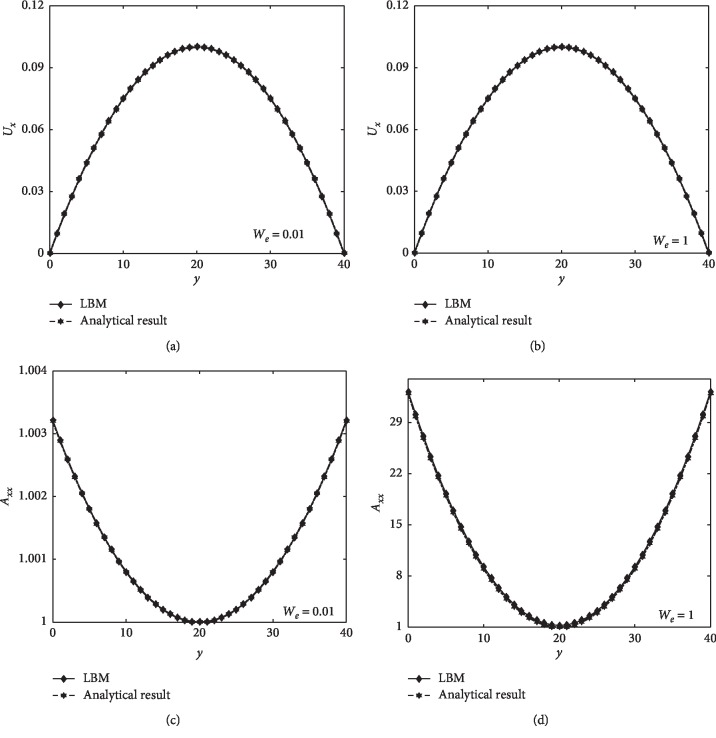
The validation results of the Oldroyd-B model. (a, b) the velocity profile at the middle cross section of the channel. (c, d) the configuration tensor component *A*_*xx*_ at the middle cross section of the channel.

From Figure 4, it can be concluded that numerical and analytical results are very close at *W*_*i*_=0.01 and 1. This indicates that our model can simulate the viscoelastic flow correctly.

## 4. Results and Discussion

The relevant parameters are set as follows. The side length of the grid of LBM is ∆*x* = ∆*y* = 1, corresponding to 0.1 *μ*m in the physical unit. The size of the channel is 600∆*x* × 102∆*y*. The Reynolds number Re = 0.05. The phase difference of the adjacent cilia is 0.02T (T is the beating cycle of cilium). The depth of PCL is fixed at 6.2 *μ*m [32]. The viscosity ratio of ML to PCL is 40. For the flow in ML, the Weissenberg number *W*_*e*_=0.01, and the viscosity ratio of the Newtonian part to the total viscosity is *η*_*M*,*N*_/(*η*_*M*,*N*_+*η*_*M*,*E*_)=0.975 [32]. The cilia beat frequency is 20 Hz [33]. The total simulation time is set as 30T, where T is the beating cycle. The cilium is structured by the node-spring model, and the default spacing of the adjacent nodes is ∆*s* = 1. The length of the cilium is *L* = 55∆*s* (5.5 *μ*m); its beat frequency is set to be 20 Hz [33].

The extensional coefficient *K*_s_ in equation (2) is set as 2.0 in nondimensional unit. This can restrict the stretching rate within the range of ±1% in simulation. The bending coefficient *K*_b_ in equation (3) is set as 1.0; this can make the cilium have certain bending rigidity and then perform a stable beating. The extensional coefficient *K*_sl_ for the elastic force *F*_c_ is set as 1.0, which can make the actual cilium move with the virtual cilium synchronously. For the membrane to divide the ML and PCL, it has the same settings of *K*_s_ and *K*_b_ to the cilium.

Based on the above settings, the beating amplitude was regulated at the case that the distance between two adjacent cilia is 0.55L [16], where we obtained the mean velocity of ML to be about 42 *μ*m/s, which is in good agreement with the results of other studies in the literature [32, 34].

### 4.1. Effect of the ML Thickness and Cilia Density

**Figure 5:**
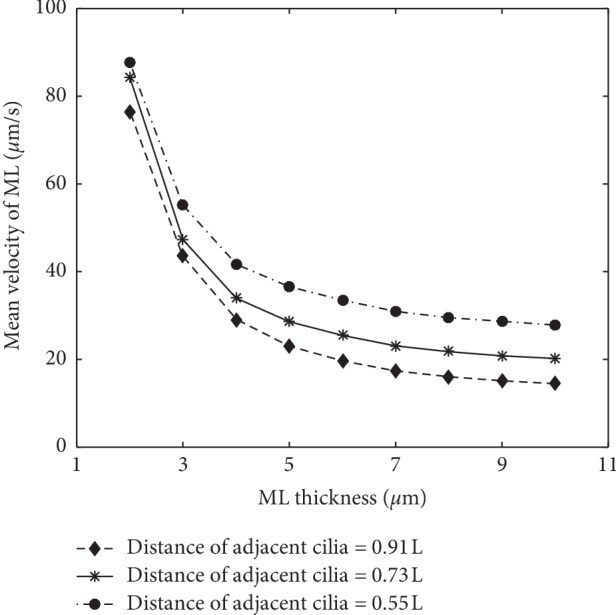
The mean velocity of ML by changing its thickness and the cilia density.

For the single PCL layer model in Reference [13], the effect of PCL depth on the transport efficiency has been discussed. It is known that if the depth of PCL increases, the transport velocities of the stream will increase at a decreasing rate. In the present study, a double-layer (PCL and ML) model is applied; it has been found that the transport efficiency is different for each layer. Here, we fix the depth of PCL as 6.2 *μ*m and then change the thickness of ML and the density of the cilia, based on which the transport velocity of ML is studied. To conduct the simulation, a thickness sequence of ML is set from 2 to 10 *μ*m with an interval of 1 *μ*m. Meanwhile, the density of the cilia is regulated by controlling the distance of two adjacent cilia, where three patterns are set as 0.55L, 0.73L, and 0.91L. The results are shown in Figure 5.

As shown in Figure 5, it is found that, in the three patterns of cilia density, the mean mucus velocity decreases as the ML thickness increases. However, in the single-layer model, the transport velocity will also increase if the depth of PCL is increased [13]. We think that the primary reason for this phenomenon is the effect of the viscoelastic fluid in ML. Two detailed reasons are analyzed below. First, the viscosity of the flow in ML is much higher than that of PCL, and the flow has less flowability. Second, the elastic property of the fluid can also affect the flow state in ML, which makes the fluid have some characteristics of the solid. This can hinder the transport of ML.

**Figure 6:**
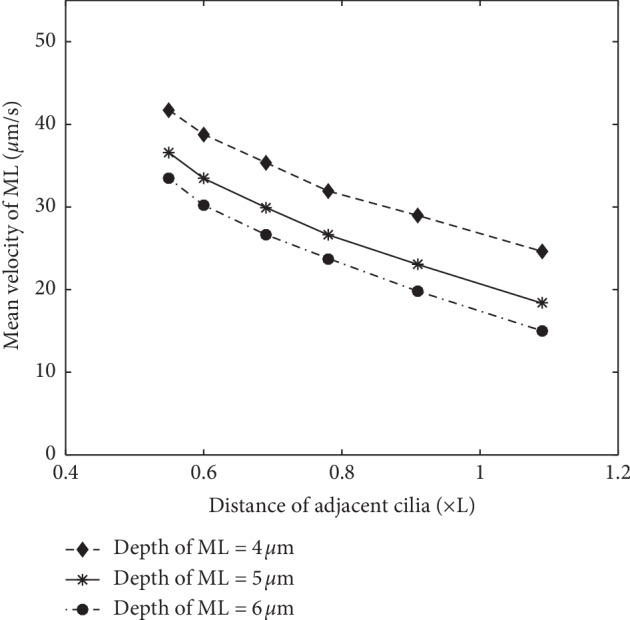
The mean velocity of the ML by changing cilia density.

In addition, it is also found that, by increasing the cilia density (decreasing the distance between two adjacent cilia), the transport of ML will accelerate. That is, a higher density can result in faster transport of ML. This is qualitatively in accord with the results from Reference [11]. In order to exhibit more details about the transport velocity under different cilia density, a distance sequence between two adjacent cilia is set as 1.09L, 0.91L, 0.78L, 0.69L, 0.6L, and 0.55L. And three patterns of ML thickness, 4 *μ*m, 5 *μ*m, and 6 *μ*m, are also considered. The results are shown in Figure 6.

These simulation results show that if the ciliary density is decreased, the velocity of the ML will also decrease. Moreover, the cilia density has an almost linear relationship with the mean velocity of the ML. This implies that smoking-induced ciliary dysfunction and even ciliary death may hinder the excretion of foreign materials by the mucociliary clearance system.

### 4.2. Effect of the Phase Difference

**Figure 7:**
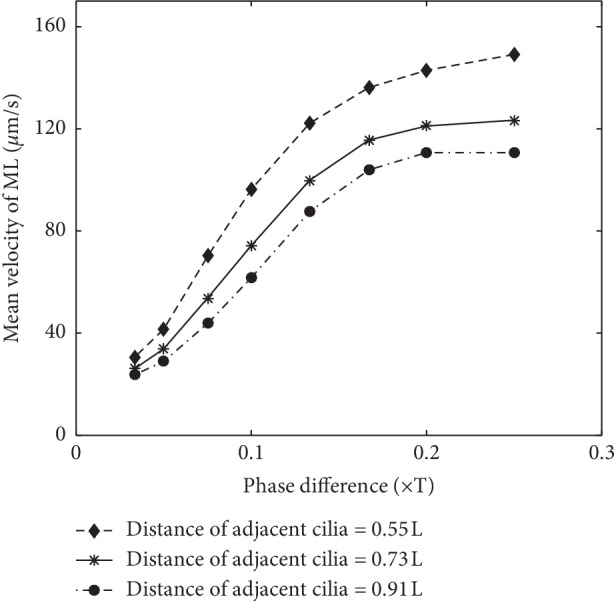
The mean velocity of ML by changing the phase difference.

We know, in most cases, the cilia beat asynchronously, where there is a phase difference between the adjacent cilia. However, how this phase difference works on the transport efficiency of ML is not clear now; it deserves a further study. In this section, a sequence of phase difference between adjacent cilia is set to study the transport efficiency of ML, 0.03T, 0.05T, 0.075T, 0.1T, 0.133T, 0.167T, 0.2T, and 0.25T. Meanwhile, three cases of cilia density are also taken into account, with a distance sequence of 0.55L, 0.73L, and 0.91L between two adjacent cilia. Worth noting is that our channel is periodic; the first beating cilium at the left inlet must keep a proper phase difference with the one at the right outlet. To meet this condition, the length of the channel must be lengthened or shortened to some extent. This operation will not influence the transport efficiency of ML because the number of cilia within a unit length of the channel is constant. The results are shown in Figure 7.

From Figure 7, we know that, in the scope of 0 to 0.25T, the velocity of the ML tends to increase by raising the phase difference. In the ranges of 0.03T to 0.05T and 0.133T to 0.25T, the increasing trend is relatively slow, and from 0.05T to 0.133T, the increasing trend is relatively fast. This indicates that the synchronous beating of the cilia is not advantageous to the transport of ML, and certain phase differences can promote transport efficiency. Actually, it can be deduced that if the phase difference is continuously raised to more than 0.25T, the transport efficiency will not be enhanced anymore.

### 4.3. Particle Migration in PCL

In general, some floating particles can be found in MCC. They may be the foreign matter from the air or some fragments from the respiratory tract surface. We know that the flow in ML has less flowability than that in PCL, so the movement of the particle in the ML is relatively simple, where the particle tends to float with the flow of the ML. In contrast, in PCL, the flow has lower viscosity and more flowability; furthermore, driven by beating cilia, this can generate a more complex flow, and the particles tend to move in complicated patterns. In such a case, to study the movement law of these particles can help us understand the clearance mechanism of the impurity in the respiratory tract.

**Figure 8:**
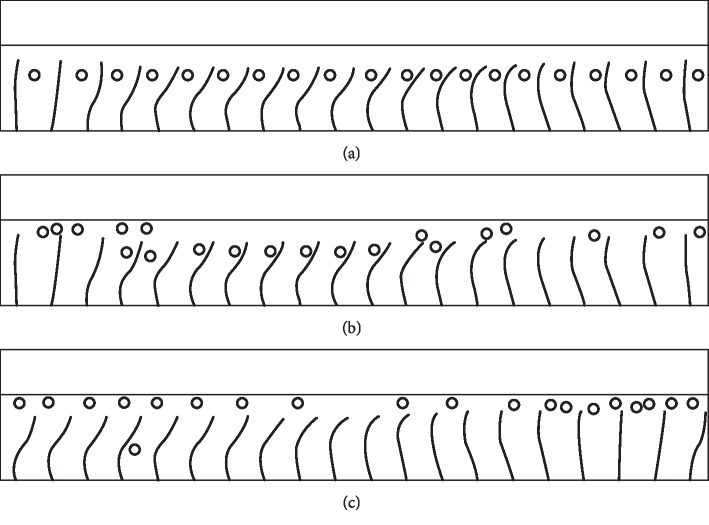
Snapshots of the distribution of particles in PCL. (a) *t* = 0. (b) *t* = 2T. (c) *t* = 20T.

In the present study, 20 hard particles were placed in PCL, where the diameter of the particle was set as 0.4 *μ*m. Their initial distribution is shown in Figure 8(a). They are at a *y*-coordinate of 4.5 *μ*m away from the bottom. With the time passing on, the snapshots of the particle distribution at *t* = 2T and 20T are picked up and displayed, respectively, in Figures 8(b) and 8(c). As a result, it has been found that almost all particles move to the top area of these cilia and get close to the ML. This indicates that the beating cilia can lead to a targeted motion of these particles, in which the particles may be captured by ML.

Furthermore, a more detailed study was conducted by varying the size and the initial position of these particles. Here, three particle diameters of 0.4 *μ*m, 0.6 *μ*m, and 0.8 *μ*m are considered for simulation. Meanwhile, the initial *y*-coordinates are set, respectively, as 2 *μ*m, 2.5 *μ*m, 3 *μ*m, 3.5 *μ*m, 4 *μ*m, 4.5 *μ*m, 5 *μ*m, and 5.5 *μ*m. In each case, 20 hard particles are placed with the distribution like that in Figure 8(a), and the mean *y*-coordinate of all these particles is set to express the overall trajectory of particle motion. The total simulation time is set as 50T.

**Figure 9:**
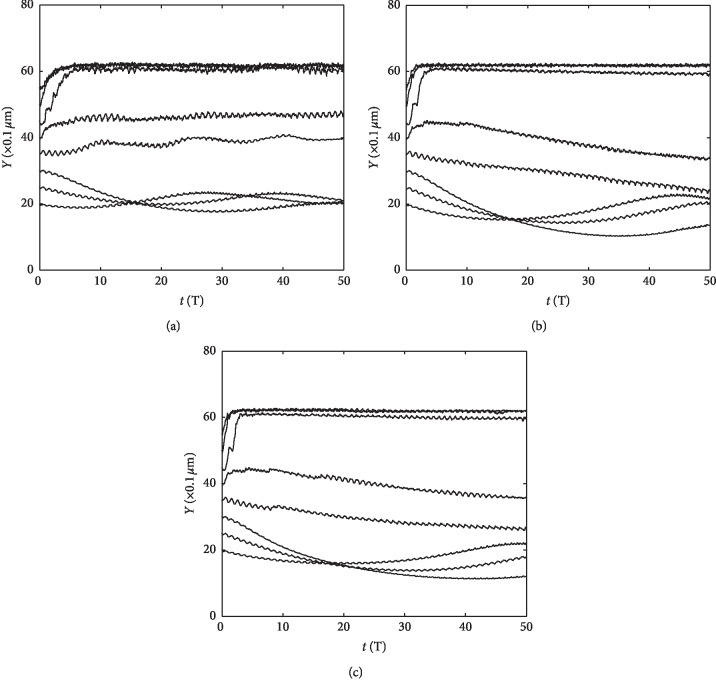
The mean *y*-coordinates of particles in PCL. (a) Particle diameter: 0.4 *μ*m. (b) Particle diameter: 0.6 *μ*m. (c) Particle diameter: 0.8 *μ*m.

The results are shown in Figures 9(a)–9(c), from which we know that, for the three types of particles in the different sizes, if the initial *y*-coordinate is larger than 4.5 *μ*m (including 4.5 *μ*m), the particles tend to migrate to the top area of the beating cilia and get close to ML within 5*T*. Once the particles reach the top area of the beating cilia, they tend to move along with ML stably. In contrast, particles with other initial positions are not able to move to the top area of the cilia; they just pace up and down in a confined area between two adjacent cilia.

## 5. Conclusion

In this paper, we employed the IB-LBM framework to model a 2D human respiratory mucociliary clearance system. Based on the model, the ML thickness, cilia density, and phase difference between adjacent cilia were varied to study the transport velocity of ML. And the migration law of particles with different sizes in PCL was also investigated. According to the simulation results, two conclusions can be summarized as follows. On one hand, for the same beating pattern in a fixed depth of PCL, the mean velocity of ML will increase by reducing the ML thickness, raising the cilia density, or enlarging the phase difference between adjacent cilia within a certain range. On the other hand, in PCL, the migration law of hard particles with different sizes is related closely to their initial position. Particles initially located on the upper part of the cilia tend to migrate upward and get close to the ML. In summary, the simulation results are reasonable, and our study can help people further understand the physical and physiologic function of the human respiratory system.

## Data Availability

The data used to support the findings of this study are available from the corresponding author upon request.
